# Longitudinal investigation of DNA methylation changes preceding adolescent psychotic experiences

**DOI:** 10.1038/s41398-019-0407-8

**Published:** 2019-02-04

**Authors:** Susanna Roberts, Matthew Suderman, Stanley Zammit, Sarah H. Watkins, Eilis Hannon, Jonathan Mill, Caroline Relton, Louise Arseneault, Chloe C. Y. Wong, Helen L. Fisher

**Affiliations:** 1 King’s College London, Social, Genetic & Developmental Psychiatry Centre, Institute of Psychiatry, Psychology & Neuroscience, London, UK; 2 University of Bristol, MRC Integrative Epidemiology Unit, Population Health Sciences, Bristol Medical School, University of Bristol, Bristol, UK; 3 University of Bristol, School of Medicine, Centre for Academic Mental Health, Bristol, UK; 4 Cardiff University School of Medicine, MRC Centre for Neuropsychiatric Genetics and Genomics, Cardiff, UK; 5 University of Exeter Medical School, University of Exeter, Exeter, UK

## Abstract

Childhood psychotic experiences (PEs), such as seeing or hearing things that others do not, or extreme paranoia, are relatively common with around 1 in 20 children reporting them at age 12. Childhood PEs are often distressing and can be predictive of schizophrenia, other psychiatric disorders, and suicide attempts in adulthood, particularly if they persist during adolescence. Previous research has demonstrated that methylomic signatures in blood could be potential biomarkers of psychotic phenomena. This study explores the association between DNA methylation (DNAm) and the emergence, persistence, and remission of PEs in childhood and adolescence. DNAm profiles were obtained from the ALSPAC cohort at birth, age 7, and age 15/17 (*n* = 901). PEs were assessed through interviews with participants at ages 12 and 18. We identified PE-associated probes (*p* < 5 × 10^−5^) and regions (corrected *p* < 0.05) at ages 12 and 18. Several of the differentially methylated probes were also associated with the continuity of PEs across adolescence. One probe (cg16459265), detected consistently at multiple timepoints in the study sample, was replicated in an independent sample of twins (*n* = 1658). Six regions, including those spanning the *HLA-DBP2* and *GDF7* genes, were consistently differentially methylated at ages 7 and 15–17. Findings from this large, population-based study suggest that DNAm at multiple stages of development may be associated with PEs in late childhood and adolescence, though further replication is required. Research uncovering biomarkers associated with pre-clinical PEs is important as it has the potential to facilitate early identification of individuals at increased risk who could benefit from preventive interventions.

## Introduction

Psychotic experiences (PEs), such as hearing voices, seeing visions, or being extremely paranoid, are experienced by around 1 in 20 children^1^, usually without a diagnosable illness. In most children these experiences remit, but nonetheless they are associated with later psychiatric problems such as psychotic disorders^2^, including schizophrenia^3,4^, and post-traumatic stress disorder^3^, as well as suicidality and self-harm in adolescence^1,5^, and suicide attempts in adulthood^3^. The likelihood of developing a psychotic disorder^6^ or post-traumatic stress disorder^7^ is especially increased if PEs persist during adolescence. Therefore, it is important to identify as early as possible which children are at risk of developing these experiences. Uncovering biomarkers associated with the onset and persistence of pre-clinical PEs in childhood can increase our understanding of the aetiological factors accompanying the development and progression of PEs, and may in the future facilitate early identification of individuals at increased psychiatric risk, and improve targeting of preventive interventions.

Biomarkers for the emergence and persistence of PEs might be evident at the level of epigenetics, that is by any of the many cellular mechanisms capable of regulating gene expression without changes in the underlying genetic sequence. DNA methylation (DNAm) patterns have, for example, been associated with a broad range of psychiatric phenotypes^8–14^, including schizophrenia and other psychotic disorders. Research in monozygotic twin pairs discordant for a clinical diagnosis of psychosis^15^ and in case–control samples^16–22^ has identified DNAm differences associated with psychosis. Importantly, these findings were identified not only in post-mortem brain tissue from diagnosed individuals^19,21,22^, but also in peripheral samples from living patients^15–18,20^, and studies have replicated findings from peripheral samples in post-mortem tissue^15^.

To date, the majority of studies have relied on cross-sectional analyses of DNAm and psychotic disorder status. Cross-sectional studies with affected individuals are likely to be highly confounded by factors associated with the disorder, such as medication. Crucially, few epigenetic studies have utilised prospective designs to focus on pre-clinical psychotic phenomena in the general population, which are important to optimise early detection efforts. To this end, research from our group recently demonstrated specific DNAm differences at age 10 between monozygotic twin pairs discordant for psychotic symptoms at age 12 (*n* = 48;^23^). Of note, hypomethylation of the top-ranked CpG site was replicated in post-mortem brain tissue in an independent sample of schizophrenia patients compared to controls, and several of the findings of interest were located in, or near, genes previously implicated in neurodevelopment and psychiatric disorders. Furthermore, a recent study found that conversion to psychosis in young help-seeking individuals (an ultra-high risk group, *n* = 39) was associated with differential changes in DNAm, with top results occurring in pathways relevant for psychosis such as oxidative stress regulation, axon guidance and inflammatory pathways^24^. These studies indicate that the PEs in pre-diagnosis populations may be associated with differential DNAm, and that the pattern of PEs may correspond with changes in DNAm in relevant pathways. However, it is difficult to draw firm conclusions based on such small samples that may not be representative of the broader population.

The aim of this study was to explore associations between genome-wide DNAm patterns and the emergence and persistence of PEs in childhood and adolescence utilising a longitudinal design covering key points in development in a large, population-based sample. First, we investigated the association between DNAm at specific timepoints during childhood and adolescence (birth, age 7, and age 15–17) and reports of PEs at ages 12 and 18. Second, we examined the role of DNAm in the continuity of PEs by comparing individuals whose experiences persisted between 12 and 18 years with those whose experiences remitted, emerged for the first time during adolescence, and those with no history of such experiences. Third, we explored the association between longitudinal trajectories of DNAm and PEs across early development. Fourth, we investigated differentially methylated regions (DMRs) associated with PEs. Finally, we tested the robustness of these findings in an independent sample.

## Methods

### Sample

The Avon Longitudinal Study of Parents and Children (ALSPAC) is a large, prospective cohort study that recruited 14,541 pregnant women resident in Avon, UK with expected dates of delivery between 1 April 1991 and 31 December 1992^25,26^. Further details of the study and available data are provided on the study website through a fully searchable data dictionary (http://www.bris.ac.uk/alspac/researchers/data-access/data-dictionary/). The current study focusses on the Accessible Resource for Integrated Epigenomic Studies (ARIES) sub-study, which consists of 1018 mother–offspring pairs who provided DNA samples at multiple timepoints^27^. All data are available by request from the Avon Longitudinal Study of Parents and Children Executive Committee (http://www.bristol.ac.uk/alspac/researchers/access/) for researchers who meet the criteria for access to confidential data.

Written informed consent was provided by parents and assent by children during the childhood phases of the study, and then by the children themselves at age 18. Ethical approval for the study was obtained from the ALSPAC Law and Ethics Committee and the Local Research Ethics Committees. Ethical approvals are in place for all sources of biological samples and data in ARIES in accordance with the Declaration of Helsinki.

### Measures

#### Psychotic experiences

PEs at ages 12 and 18 were assessed via semi-structured interviews (Psychosis-like symptoms semi-structured interview—PLIKSi) that have been described previously^2,28^. At 12 years participants were asked about PEs over the previous 6 months; at 18 years participants were asked about experiences since the age of 12. Further details regarding determination of PEs in this sample are provided in the Supplementary Information. For longitudinal analyses, individuals were included if they had PE data available at both timepoints, and the nature of PEs from ages 12 to 18 was determined as persistent (present at both timepoints), remitted (present at 12 but not 18), emergent (present at 18 but not 12), and none (no experiences at either timepoint).

#### DNA methylation

DNA samples were extracted from cord blood on delivery, and from peripheral blood samples in childhood (age 7) and in adolescence (age 15–17) according to established procedures^27^. DNA was bisulfite-converted using the Zymo EZ DNA MethylationTM kit (Zymo, Irvine, CA) and then DNA methylation of over 485,000 CpG sites was quantified using the Illumina Infinium HumanMethylation450K BeadChip assay (HM450; Illumina Inc., CA). Arrays were scanned using the Illumina iScan, and GenomeStudio (version 2011.1; Illumina Inc.) was used to extract signal intensities and assess initial quality review.

#### Pre-processing and QC procedures

HM450 detects the proportion of molecules methylated at each CpG site on the array. For each sample, the estimated methylation level at each CpG site is expressed as a beta value (*β*), which is the ratio of the methylated probe intensity to the overall intensity and ranges from 0 (no cytosine methylation) to 1 (complete cytosine methylation). Background correction and functional normalisation were performed using meffil^29^. Samples with >10% of sites with a detection *p* value >0.01 or a bead count <3 in >10% of probes were removed from further analysis. Non-specific probes and probes on sex chromosomes were removed^30,31^. Following QC procedures, data were available for 381,871 probes. Probes were annotated using information provided by Illumina (genome build: hg19).

#### Batch effects

Samples from all timepoints in ARIES were distributed across slides using a semi-random approach, with all samples from each individual presented on the same array to minimise confounding by batch. Further batch variables were recorded using a purpose-built laboratory information management system, which also recorded QC metrics from the standard control probes on the HM450 array. Samples failing QC (>20% probes with *p* ≥ 0.01) were repeated and, if unsuccessful, excluded from further analysis.

#### Cell heterogeneity

To account for potential differences in methylation arising from cell composition in whole-blood samples, cell counts were estimated using the Houseman algorithm^32^ and included as covariates. However, for longitudinal analyses including data from cord blood and whole-blood samples, analyses instead included the first 20 independent surrogate variable components to account for both heterogeneity between cord blood and peripheral blood samples as well as batch effects. Previous research has indicated that surrogate variables derived in this way account for cell count heterogeneity as well as estimated cell counts^33,34^.

#### Potential confounders

Pregnancy and birth-related variables were considered as potential confounders, including parity (number of pregnancies resulting in live birth), maternal age at child’s birth (derived from date of birth), maternal smoking during pregnancy (never, stopped first trimester, continued throughout pregnancy), infections during pregnancy (at any time), and delivery method (caesarean; yes/no). Factors relating to the child included gender, smoking, and use of psychiatric medications. Data concerning child smoking behaviour during adolescence were not available for all samples; therefore, a proxy smoking variable was derived using DNAm signals from probes previously associated with smoking^35,36^. This method has been shown to distinguish between smokers and non-smokers (e.g. see ^20^). One child reported use of psychiatric medications across the study period and was removed from the analysis sample.

### Statistical analyses

We investigated the association between DNAm and PEs in four stages. All analyses were conducted using R (version 3.3.1) unless otherwise stated.

#### Epigenome-wide Association Study

The Epigenome-wide Association Study (EWAS) function in meffil^29^ was used to conduct six epigenome-wide association studies of PEs at ages 12 and 18 with cord blood, age-7 peripheral blood, and age-15/17 peripheral blood DNAm. Parity, maternal age, maternal smoking during pregnancy, infections during pregnancy, delivery methods, and child’s gender were included as covariates, as well as estimated cell counts. Analyses using DNAm data from age 7 or age 15–17 also included child age as a covariate. Probes with values at the extremes of the distribution (5%) were winsorised. Statistical significance was determined using a Bonferroni correction, giving a threshold of *p* < 1.3 × 10^−7^. Tests with *p* < 5 × 10^−5^ were defined as reaching suggestive significance.

#### Continuity of PEs across adolescence

The CpG sites most strongly associated with PEs at either timepoint (12 or 18, *p* < 5 × 10^−5^) were assessed for association with the continuity of PEs between 12 and 18 (persistent, remitted, emergent, none) using ANOVA. Post hoc tests were used to determine the relationships between groups. Significant results (*p* < 0.00045; *p* = 0.05/110 CpG sites) were re-run with linear models to include all previously described covariates.

#### Longitudinal DNAm trajectories and continuity of PEs

Multilevel models were constructed to test the association between the methylomic trajectories of the top CpG sites and the continuity of PEs across adolescence, where DNAm was the dependent variable. A linear spline term with a knot at 7 was included to allow for different linear changes from 0 to 7 and from 7 to 15–17. Models were also adjusted for all previously described pregnancy and birth-related covariates, and the first 20 independent surrogate variable components to account for heterogeneity between cord blood and peripheral blood samples (e.g. cell composition and batch effects). Models were repeated including derived smoking scores as previously described.

#### Differentially methylated regions

The *comb-p* module in Python was used to identify DMRs by combining spatially correlated *p* values from each EWAS. We used a seed *p* value of <0.001, a maximum distance of 500 bp, and a minimum of three probes. Statistical significance was determined using a Šidák correction for multiple testing, where *p* < 0.05 is significant.

#### Gene Ontology (GO) enrichment analysis

Enrichment analyses were conducted to assess the over-representation of GO^37^ biological processes and functions in CpG sites of each EWAS where *p* < 0.001. A lenient significance threshold was used to maximise our probes of interest. We used a methodology that controls for the number of probes that are annotated to each gene, as well as taking into account the hierarchical structure of ontological categories by grouping terms where the significant enrichment was explained by the overlap with a more significant term (as described in ref. ^20^).

#### Replication

CpG sites of interest (detailed below) were tested for association with PEs in the Environmental Risk (E-Risk) Longitudinal Twin Study, a nationally representative cohort of 2232 British-born twins, which has been previously described in detail^38^. In this sample, DNAm from whole-blood samples obtained at age 18 was also quantified using the Illumina Infinium HumanMethylation450K BeadChip assay (HM450; Illumina Inc., CA)^39^, and PEs between 12 and 18 years were obtained from an interview that is similar to the PLIKSi^40^. Following stringent quality control procedures using the wateRmelon package^41^, data were available for 1658 individuals at age 18. The associations between DNAm at our CpG sites of interest and concurrent PEs at age 18 were tested using linear regression, controlling for gender, zygosity, batch, and cell counts (determined using the Houseman algorithm as previously described). Clustered standard errors were used to calculate *p* values to account for the effect of family, and were implemented using the plm package^42^. For the closest consistency between timepoints in the two studies, CpG sites associated with PEs at age 18 (*p* < 5 × 10^−5^) in the age 15–17 DNAm data from ALSPAC were tested in the E-Risk sample. Consistently detected CpG sites, that is, those detected in the top 100 most significant results of more than one EWAS in ALSPAC, were also tested in the E-Risk sample.

## Results

### Sample characteristics

DNAm data were available on 901 samples from cord blood, 966 at age 7, and 966 at age 15–17 (*n* = 845 with all three). In those with at least one DNA sample (*n* = 999), 978 had PE data available for at least one timepoint (age 12: *n* = 929; age 18: *n* = 816). At age 12, 12.5% (*n* = 116) reported PEs. At age 18, 8.8% (*n* = 72) reported PEs. In total, 767 individuals had DNAm and PE data available for all timepoints. Of those, 81.4% had no PEs at either timepoint; 10.2% had PEs that remitted between 12 and 18; 6.2% had PEs that emerged between 12 and 18; and 2.2% had PEs that were persistent between 12 and 18. Sample characteristics are displayed in Table 1.

**Table 1:** Sample descriptives

		DNA methylation from cord blood	DNA methylation at age 7	DNA methylation at age 17
	PEs at age 12 (*n*)	833	900	899
	PEs at age 18 (*n*)	739	791	787
	**Characteristic**	***n***	**%**	**(missing** ***n*** **)**
	PEs at age 12	116	12.5	69
	PEs at age 18	72	8.8	182
	*Parity*			35
	0	451	46.8	
	1	358	37.2	
	2	117	12.1	
	3	37	3.8	
	Maternal age	Mean = 29.5	sd = 4.4	6
	*Maternal smoking*			18
	Never	847	86.4	
	Stopped during pregnancy	36	3.7	
	Continued throughout pregnancy	97	9.9	
	Infections during pregnancy	445	44.6	
	Caesarean section delivery	92	9.6	40
	Sex (male)	485	48.6	

*PEs* psychotic experiences

### DNAm and PEs—overview

Differences in DNAm at birth, age 7, and ages 15–17 between those with and without PEs at ages 12 and 18 did not survive correction for multiple testing (*p* < 1.3 × 10^−7^; reflecting 381,871 sites in each analysis). However, a number of methylation probes were nominally significant in each analysis (*p* < 5 × 10^−5^, detailed in the Supplementary Information). The top 20 differentially methylated positions (DMPs) for each analysis are detailed in Table 2a–f, with a more extensive list provided in Supplementary Tables (top 100 DMPs: Tables S1–S6). Comparison of the top 100 DMPs across analyses showed three CpG sites (cg16459265, cg24940155, cg25184754) detected in more than one analysis and with the same direction of effect, and nine genes that were detected in more than one analysis but with different CpG sites (Supplementary Table S7).

**Table 2a:** Cord blood methylation and age-12 psychotic experiences: top 20 probes

Probe	*p* value	*t* statistic	Chromosome	Annotated gene
*cg20862283*	8.66E-06	*4.48*	*chr2*	
*cg10490202*	*1.21E-05*	*−4.4*	*chr17*	*RPTOR*
*cg21040096*	*2.15E-05*	*4.27*	*chr17*	*RPH3AL*
*cg18752363*	*2.21E-05*	*−4.27*	*chr4*	*C4orf10;NOP14*
*cg11271415*	*3.15E-05*	*4.19*	*chr8*	*FBXO32*
*cg20083186*	*4.06E-05*	*4.13*	*chr3*	
*cg00712792*	*4.28E-05*	*−4.11*	*chr16*	*SPIRE2*
cg11936556	5.14E-05	4.07	chr5	
cg14892222	5.15E-05	4.07	chr15	GOLGA8A
cg26672652	5.25E-05	4.07	chr8	PXDNL
cg13751188	5.26E-05	−4.07	chr11	TECTA
cg14829814	5.39E-05	4.06	chr12	
cg26302009	6.39E-05	4.02	chr6	PHACTR1
cg01840115	6.94E-05	−4	chr13	
cg06893379	7.29E-05	3.99	chr5	
cg04409048	7.41E-05	3.98	chr6	
cg08105378	7.57E-05	−3.98	chr19	HOMER3
cg07438586	7.92E-05	−3.97	chr6	GNL1
cg04114269	8.76E-05	−3.94	chr3	C3orf26;FILIP1L;MIR548G

Italic rows indicate *p* < 5 × 10^−5^

**Table 2b:** Cord blood methylation and age-18 psychotic experiences: top 20 probes

Probe	*p* value	*t* statistic	Chromosome	Annotated gene
*cg00407329*	*1.84E-05*	*4.31*	*chr6*	*SIM1*
*cg17972930*	*2.76E-05*	*4.22*	*chr15*	*USP50*
*cg07571954*	*3.22E-05*	*−4.18*	*chr10*	*C10orf131*
*cg05138918*	*4.56E-05*	*−4.1*	*chr3*	
*cg17843418*	*4.64E-05*	*−4.1*	*chr17*	*TBC1D16*
cg21167905	8.29E-05	3.96	chr1	C1orf9
cg09825080	8.67E-05	−3.95	chr16	
cg02424103	9.58E-05	3.92	chr3	
cg16426764	0.0001	−3.9	chr10	LHPP
cg00680277	0.00012	3.87	chr2	TTC15
cg23463608	0.00012	−3.86	chr19	GNG7
cg25930186	0.00012	−3.86	chr20	MOCS3;DPM1
cg01395281	0.00013	−3.86	chr1	ZNF593
cg25275750	0.00015	3.82	chr13	TBC1D4
cg16327647	0.00016	−3.8	chr1	CENPF
cg01496136	0.00017	−3.78	chr16	
cg09568647	0.00017	−3.77	chr5	
cg22694818	0.00018	3.77	chr1	LHX8
cg06475223	0.00018	−3.76	chr18	LAMA3
cg12859112	0.00019	3.76	chr6	TMEM14B

Italic rows indicate *p* < 5 × 10^−5^

**Table 2c:** Age-7 methylation and age-12 psychotic experiences: top 20 probes

Probe	*p* value	*t* statistic	Chromosome	Annotated gene
*cg22499215*	*2.97E-07*	*−5.16*	*chr22*	
*cg14263553*	*1.12E-06*	*−4.9*	*chr8*	
*cg07366553*	*2.35E-06*	*−4.75*	*chr22*	*SELO*
*cg09553452*	*2.75E-06*	*−4.72*	*chr16*	*CDH5*
*cg02284814*	*4.82E-06*	*−4.6*	*chr1*	*PPP1R12B*
*cg17160666*	*4.95E-06*	*−4.6*	*chr10*	
*cg17139666*	*5.04E-06*	*4.59*	*chr4*	
*cg08032135*	*5.08E-06*	*4.59*	*chr8*	*NRG1*
*cg04584761*	*1.00E-05*	*−4.56*	*chr2*	*SUPT7L*
*cg10756647*	*1.00E-05*	*4.55*	*chr7*	*PSPH*
*cg16451872*	*1.00E-05*	*4.54*	*chr15*	*UBE3A*
*cg10725301*	*1.00E-05*	*−4.52*	*chr6*	*ZKSCAN3*
*cg18477816*	*1.00E-05*	*−4.52*	*chr11*	*IRF7*
*cg10143301*	*1.00E-05*	*4.51*	*chr5*	*TXNDC15*
*cg02572956*	*1.00E-05*	*−4.48*	*chr7*	*CHPF2;MIR671*
*cg01040786*	*1.00E-05*	*−4.41*	*chr5*	
*cg02975060*	*1.00E-05*	*−4.4*	*chr14*	*MIR1185-2*
*cg23658045*	*1.00E-05*	*4.4*	*chr12*	*DCTN2*
*cg10151454*	*1.00E-05*	*−4.36*	*chr10*	*EBF3*
*cg10303842*	*2.00E-05*	*4.35*	*chr5*	*CDH12*

Italic rows indicate *p* < 5 × 10^−5^

**Table 2d:** Age-7 methylation and age-18 psychotic experiences: top 20 probes

Probe	*p* value	*t* statistic	Chromosome	Annotated gene
*cg00995854*	*2.74E-06*	*−4.72*	*chr1*	*CD5L*
*cg13845105*	*3.08E-06*	*−4.7*	*chr12*	*RPL6*
*cg27190398*	*8.76E-06*	*−4.48*	*chr16*	*STUB1;JMJD8*
*cg07143863*	*9.18E-06*	*−4.47*	*chr12*	
*cg10468951*	*1.33E-05*	*−4.38*	*chr3*	*C3orf45*
*cg05927274*	*1.79E-05*	*−4.32*	*chr1*	
*cg09936919*	*1.90E-05*	*−4.3*	*chr2*	
*cg15129144*	*1.95E-05*	*−4.3*	*chr2*	*EPAS1*
*cg03332469*	*2.00E-05*	*4.28*	*chr7*	
*cg02743632*	*2.00E-05*	*−4.28*	*chr1*	*HLX*
*cg20477259*	*2.00E-05*	*−4.25*	*chr6*	*TNF*
*cg24110396*	*3.00E-05*	*−4.22*	*chr10*	*CCNY*
*cg27185423*	*3.00E-05*	*−4.21*	*chr15*	
*cg08522143*	*3.00E-05*	*−4.19*	*chr19*	*ZNF14*
*cg04936274*	*3.00E-05*	*−4.18*	*chr10*	
*cg14468692*	*3.00E-05*	*−4.18*	*chr11*	*RNH1*
*cg15031103*	*4.00E-05*	*−4.15*	*chr10*	*LRRC27*
*cg05072413*	*4.00E-05*	*−4.11*	*chr22*	*FAM19A5*
*cg16459265*	*4.00E-05*	*−4.11*	*chr7*	*C7orf40;SNORA9*
cg02108135	5.00E-05	−4.06	chr17	

Italic rows indicate *p* < 5 × 10^−5^

**Table 2e:** Age 15–17 methylation and age-12 psychotic experiences: top 20 probes

Probe	*p* value	*t* statistic	Chromosome	Annotated gene
*cg14284469*	*1.87E-06*	*4.8*	*chr8*	*STMN2*
*cg00956759*	*2.94E-06*	*−4.71*	*chr17*	
*cg12009697*	*4.13E-06*	*−4.63*	*chr2*	
*cg21602768*	*8.15E-06*	*−4.49*	*chr20*	*ABHD12*
*cg17027353*	*1.25E-05*	*4.39*	*chr6*	
*cg14386808*	*1.34E-05*	*−4.38*	*chr8*	
*cg22035229*	*1.77E-05*	*−4.32*	*chr1*	*MSH4*
*cg16817992*	*2.11E-05*	*4.28*	*chr7*	*KCNH2*
*cg25344401*	*3.26E-05*	*4.18*	*chr7*	*FOXK1*
*cg05942128*	*4.71E-05*	*4.09*	*chr2*	*HOXD11*
cg26370729	5.19E-05	4.07	chr5	F2RL1
cg25024734	5.84E-05	4.04	chr10	
cg03945301	6.32E-05	−4.02	chr6	EHMT2
cg17103467	7.84E-05	−3.97	chr1	WDR8
cg24695977	7.92E-05	3.97	chr1	
cg04554240	8.12E-05	−3.96	chr3	ESYT3
cg08621773	1.02E-04	−3.9	chr2	
cg27280332	1.04E-04	−3.9	chr12	
cg25827710	0.00011	−3.88	chr13	LCP1
cg15318176	0.00012	−3.86	chr7	PTPRN2

Italic rows indicate *p* < 5 × 10^−5^

**Table 2f:** Age 15–17 methylation and age-18 psychotic experiences: top 20 probes

Probe	*p* value	*t* statistic	Chromosome	Annotated gene
*cg25975712*	*1.13E-05*	*4.42*	*chr22*	*FAM19A5*
*cg24177611*	*1.15E-05*	*−4.42*	*chr10*	*KNDC1*
*cg25324164*	*1.57E-05*	*−4.35*	*chr11*	*FADS2*
*cg15986671*	*4.00E-05*	*−4.13*	*chr7*	
*cg16073378*	*4.74E-05*	*4.09*	*chr2*	
*cg04942547*	*4.82E-05*	*−4.09*	*chr20*	*ZFP64*
cg11333576	5.26E-05	−4.07	chr19	SHC2
cg16459265	5.51E-05	−4.06	chr7	C7orf40;SNORA9
cg09222367	7.68E-05	−3.98	chr15	IQGAP1
cg23415756	1.02E-04	3.91	chr17	NTN1
cg18656829	1.14E-04	3.88	chr13	
cg06046431	1.15E-04	3.88	chr11	BDNF
cg16791444	1.17E-04	−3.87	chr17	
cg11604728	1.17E-04	−3.87	chr7	POU6F2;POU6F2
cg22012759	1.19E-04	−3.87	chr1	
cg19052355	1.25E-04	3.86	chr2	GBX2
cg05580655	1.30E-04	3.85	chr3	SCHIP1
cg16740157	1.43E-04	−3.82	chr6	
cg26530497	0.000156178	−3.8	chr13	SOHLH2
cg19769301	0.000156717	3.8	chr1	

Italic rows indicate *p* < 5 × 10^−5^

### Cord blood DNAm and PEs

Analysis of cord blood DNAm identified seven nominally significant CpG sites associated with age-12 PEs (Table 2a), and five with age-18 PEs (Table 2b). The top CpG site at age 12 was cg20862283 (*p* = 8.66 × 10^-6^), which was not mapped to a RefSeq gene. GO enrichment analysis of the CpG sites where *p* < 0.001 from analysis of PEs at age 12 identified 104 groups of related GO categories (*p* < 0.05, Table S8). At age 18, the top CpG site was cg00407329 (*p* = 1.84 × 10^−5^), which was annotated with the gene *SIM1*. Fifty-one groups of GO enrichment terms were identified (Table S9). Ranked highly among the grouped terms were “Wnt-activated receptor activity” and “central nervous system neuron development”. The top-ranked terms did not show any similarities between timepoints.

### Age-7 DNAm and PEs

Analysis of age-7 DNAm identified 63 nominally significant CpG sites associated with age-12 PEs (Table 2c), and 19 with age-18 PEs (Table 2d). At age 12, the top CpG site was cg22499215 (*p* = 2.97 × 10^−7^), which was not annotated with a RefSeq gene. In the top CpG sites, 92 groups of GO terms were identified (Table S10), including a number of terms related to brain development and “*Wnt* signalling pathway”. At age 18, the top CpG site was cg00995854 (*p* = 2.74 × 10^−^^6^, *CD5L*). In GO enrichment analysis, 137 groups of terms were identified (Table S11). The top-ranked term was “proteoglycan binding”, including a number of terms related to immune processes, as well as “neurogenesis” and terms representing neuron development and projection. The top-ranked terms did not show any similarities between timepoints.

### Age-15–17 DNAm and PEs

Analysis of age-15–17 DNAm identified ten nominally significant CpG sites associated with age-12 PEs (Table 2e), and six with age-18 PEs (Table 2f). At age 12, the top CpG site was cg14284469 (*p* *=* 1.87 × 10^−6^, *STMN2)*, and 114 grouped GO terms were identified (Table S12). At age 18, the top CpG site was cg25975712 (*p* = 1.13 × 10^−5^*, FAM19A5)*, and 123 grouped GO terms were identified (Table S13). The top-ranked term was “rostrocaudal neural tube patterning”, which largely included terms related to neuronal and physical development and morphogenesis. Also within the top grouped terms were “behavioural fear response” and “behavioural defense response”. The top-ranked terms did not show any similarities between timepoints.

### DNAm from cord blood, at age 7, and at ages 15–17 and continuity of PEs between ages 12 and 18

**Fig. 1:**
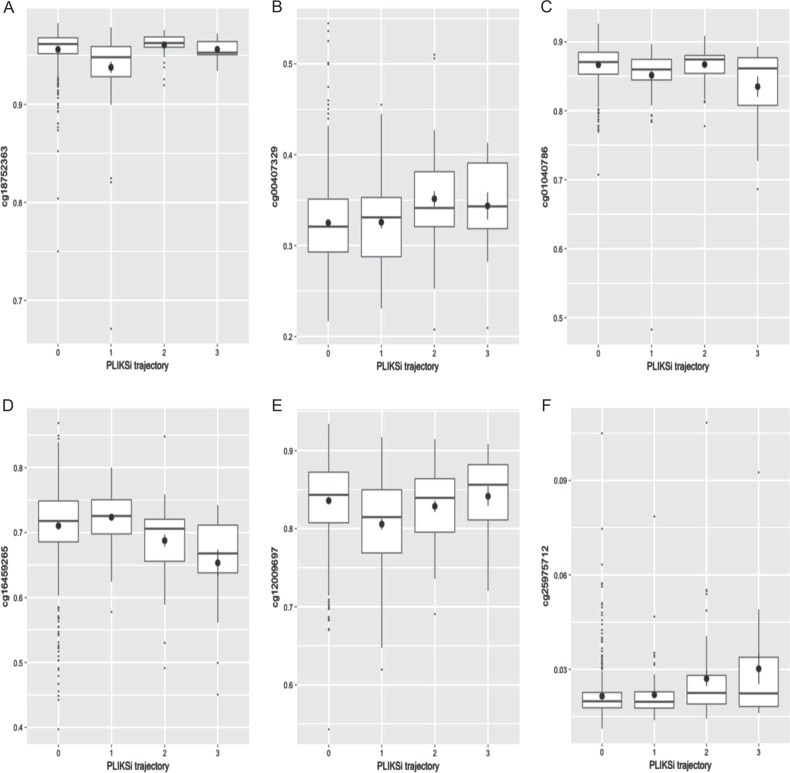
**a–f** DNA methylation and the continuity of psychotic experiences (PEs) between ages 12 and 18. Boxplots represent the most strongly associated CpG site (tested using ANOVA) at each timepoint. **a** DNA methylation from cord blood, CpG site detected at age 12; **b** DNA methylation from cord blood, CpG site detected at age 18; **c** DNA methylation at age 7, CpG site detected at age 12; **d** DNA methylation at age 7, CpG site detected at age 18; **e** DNA methylation at age 15–17, CpG site detected at age 12; **f** DNA methylation at age 15–17, CpG site detected at age 18. For each boxplot, 0 = never had PEs, 1 = PEs remitted between 12 and 18, 2 = PEs emerged between 12 and 18, and 3 = PEs persisted between 12 and 18 years. Circles represent group means. *PLIKSi* Psychosis-like symptoms semi-structured interview

Next, we tested the association between timepoint-specific DNAm and continuity of PEs across adolescence for each of the top CpG sites in each EWAS (*p* < 5 × 10^−5^). Results for these analyses are provided in the Supplementary Information, Table S14. Different patterns of DNAm at each timepoint were observed in relation to PE continuity. At some CpG sites, statistical differences between the groups were limited to one group (e.g. cg18752363, Fig. 1a). At other CpG sites, individuals whose PEs had remitted by age 18 had DNAm profiles more similar to those who never had any PEs, whereas those with persistent PEs tended to show the greatest differences in DNAm profiles, while those whose PEs emerged between 12 and 18 often had intermediary differences between the none/remitted groups and the persistent group (e.g. cg16459265, Fig. 1d; full statistical results reported in Table S14, Fig. 1a–f).

### Longitudinal DNAm changes and continuity of PEs

**Fig. 2:**
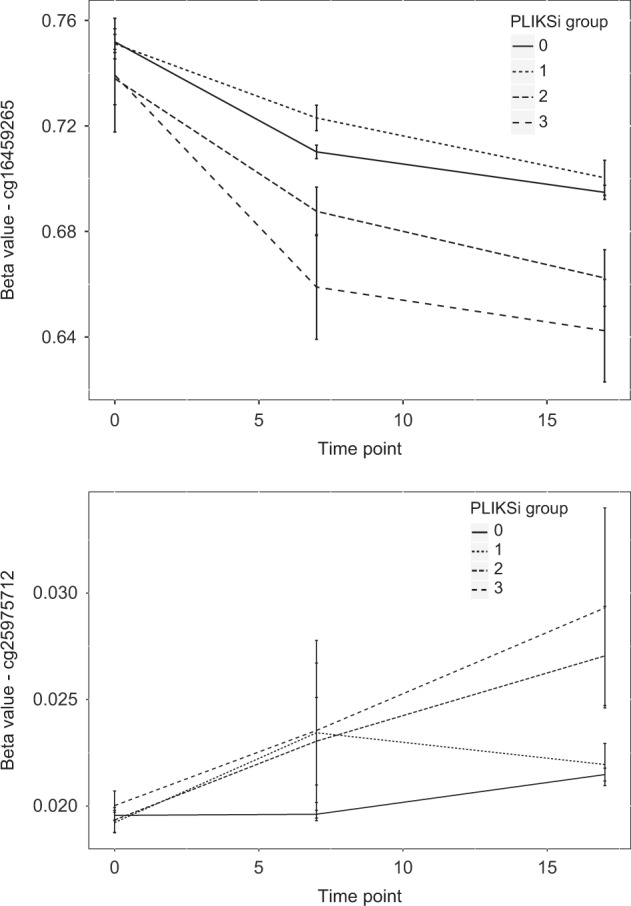
**a**, **b** DNA methylation trajectories by PLIKSi psychotic experiences (PEs) group: 0 = no PEs at either timepoint; 1 = PEs at age 12 that remitted by age 18; 2 = no PEs at age 12 but emerged by age 18; 3 = PEs that persisted from age 12 to age 18. **a** cg16459265 methylation and PEs. **b** cg25975712 methylation and PEs. Lines represent mean DNA methylation per sample timepoint; bars represent standard errors. *PLIKSi* Psychosis-like symptoms semi-structured interview

We tested the association between longitudinal DNAm and continuity of PEs between 12 and 18 for the previously identified top CpG sites. Results are provided in Supplementary Table 15 for the full longitudinal model. Longitudinal models were unaffected by the inclusion of a DNAm-derived smoking score. For several CpG sites, the methylation trajectory was nominally different between groups (remitted, emergent, or persistent PEs—examples are given in Fig. 2).

### Differentially methylated regions

We tested for DMRs using the *comb-p* algorithm. Significant DMRs (Šidák corrected *p* < 0.05) were detected at all timepoints, and are detailed in the Supplementary Information (Table S16). Nine DMRs were identified in cord blood associated with age-12 PEs; four with age-18 PEs. Thirty-five DMRs were identified in age-7 samples associated with age-12 PEs, 11 with age-18 PEs. Fourteen DMRs were identified in age 15–17 samples associated with age-12 PEs, 11 with age-18 PEs. Of note, six regions were differentially methylation in association with either age-12 or age-18 PEs in both age-7 and age 15–17 samples, spanning regions including *HLA-DPB2* and *HIVEP3* (associated with age-12 PEs), and *GDF7* (age-18 PEs). A full list of significant DMRs at each timepoint is available in the Supplementary Information, Table S16.

### Replication

No associations between DNAm at age 15–17 and PEs at age 18 in the ALSPAC sample were replicated in the E-Risk sample (all *p*’s > 0.05). However, DNAm at the CpG site cg16459265 (consistently detected among the top 100 CpG sites in the ALSPAC sample across the different ages) was found to be associated with age-18 PEs in the E-Risk sample. At this probe, individuals with any PEs at age 18 had marginally lower DNAm than those without PEs (difference = −0.76%, *p* = 0.0029) in keeping with the hypomethylation found in the ALSPAC sample.

## Discussion

### Summary

This study utilised longitudinal data in a large, population-based sample to investigate the association between epigenome-wide DNAm patterns and the emergence and persistence of PEs in childhood and adolescence. While no CpG sites reached Bonferroni-corrected significance in this study, a number of CpG sites and genes in biological pathways relevant to psychosis appeared highly ranked in these analyses. Genes detected in the top 100 CpG sites of more than one analysis (though not with the same CpG site) included *BAIAP2*, *FGFR3* and *MAD1L1*, and genetic mutations in these genes have previously been implicated in susceptibility to psychotic disorders^43–45^. Also consistently detected were *LFNG* and *LRP5*, part of the *Notch* and *Wnt* signalling pathways (respectively), which have been reported in previous studies of psychiatric phenotypes including schizophrenia^46,47^. None of the individual CpG sites associated with PEs at age 18 in the ALSPAC sample were replicated in the independent E-Risk sample. Nonetheless, attempts to replicate these results should be undertaken in other large population-based longitudinal samples. Using a DMR approach, six regions associated with PEs were detected consistently in both age-7 and age 15–17 samples. The top DMR associated with age-12 PEs at age 7 (and also significant at age 15–17) spanned 18 probes in the *HLA-DPB2*, part of the major histocompatibility complex on chromosome 6, which has been widely implicated in schizophrenia^48^. The top DMR associated with age-18 PEs in age-7 samples overlapped substantially with the top DMR in age 15–17 samples, spanning 7–9 probes on chromosome 2 at exon 2 of the *GDF7* gene. DNAm at this gene has previously been associated with early age of onset in anorexia nervosa^49^ and is thought to play a role in nervous system development.

The top CpG sites identified showed interesting patterns of change in DNAm across development according to the continuity of PEs across adolescence. Some CpG sites distinguished individuals whose PEs remitted or never had PEs from those whose experiences persisted across or emerged during adolescence. An example is CpG site cg16459265 (annotated to the RefSeq genes *C7orf40;SNORA9*), a top CpG site associated with age-18 PEs in DNA samples from both childhood (age 7) and adolescence (ages 15–17). Lower DNAm at this CpG site was also associated with PEs at age 18 in the E-Risk sample. Similarly, at the CpG site cg25975712 (*FAM19A5*), DNAm patterns diverged during childhood according to PE status, but by adolescence, those whose PEs remitted had DNAm profiles more similar to those with no PEs (though it should be noted that DNAm is generally low at this CpG site).

### Comparison to the literature

Two studies have previously investigated the association between DNAm and subclinical psychotic phenomena. However, there are some differences in the study design between the current study and this previous research. We utilised DNAm from birth to adolescence in a large, population-based, unrelated sample, whereas earlier studies were conducted in smaller samples of monozygotic twin pairs discordant for childhood psychotic symptoms^23^ or examined conversion to psychosis in ultra-high risk, help-seeking individuals^24^. Study participants also differed in age at sample collection (ages 5 and 10 in ref. ^23^; age 16–30 in ref. ^24^) and sample type (buccal swabs in ref. ^23^). The differences in time periods are an important consideration when comparing these studies, as there is little concurrence. The current study considers a broad span of ages, compared to shorter intervals or more specific timepoints reported in the previous reports. While the ages included in this study cover important timepoints developmentally, it is not possible to accurately infer what is happening in the intervening years, or identify changes in DNAm accompanying the specific period that the PEs were measured. As such, it is perhaps unsurprising that none of the CpG site associations detected here are seen in either previous study. Furthermore, we do not have information regarding psychotic phenomena in the years following those included in this study. Nevertheless, associations linked to a small number of genes are observed across the studies. One example is the gene *PTPRN2*, which is thought to be necessary for normal neurotransmitter activity in the brain. Of note, the DMR located on chromosome 6 (and annotated to the gene *HLA-DPB2*) which was associated with age-12 PEs in our study substantially overlaps with a similar DMR detected in prefrontal cortex samples associated with polygenic risk for schizophrenia^50^. Taken together, these studies provide tentative early evidence for potential methylomic changes accompanying the development of early psychosis-related phenomena, albeit with some inconsistency regarding the location of these changes.

### Methodological considerations

This study has a number of strengths. Analyses were conducted in the relatively large ARIES sample drawn from a population-based cohort study, with DNAm profiled at multiple timepoints across early development and PEs assessed in childhood and adolescence. Prospective collection of high-quality phenotypic data in this cohort allowed for investigation of the temporal relationship between DNAm and PEs and repeated assessments enabled investigation of trajectories of methylation and continuity of PEs. Extensive data regarding potential confounding factors were also available and included in the analyses. Furthermore, this is the largest study to date to examine the association between DNAm and pre-clinical PEs. Research investigating DNAm in psychotic disorders is typically performed in adult samples, where the confounding effects of medication use are difficult to avoid. As ARIES is a young, population-based sample, only one participant was using psychiatric medication (and was excluded from these analyses). Additionally, the longitudinal investigation of early pre-clinical psychotic experiences is particularly important to better understand biological factors associated with the aetiology of psychotic and other psychiatric disorders and facilitate early identification of those at risk.

However, there are methodological factors that should be taken into consideration when interpreting these results. Firstly, DNAm data were generated from peripheral samples (cord blood and whole blood). This approach is necessary when conducting research in large longitudinal samples (i.e. with live participants), where the most relevant tissue (brain) is not available. Previous research has demonstrated tissue specificity in DNAm patterns^51^, limiting the conclusions that can be drawn from studies performed in blood. However, comparison of DNAm from blood and brain samples is possible in reference datasets. For example, for the CpG site cg16459265, although DNAm in blood samples is lower and shows more variation than in brain tissue, DNAm in blood is strongly correlated with DNAm in matched samples of prefrontal cortex, entorhinal cortex, superior temporal gyrus, and cerebellum^52^. Nevertheless, for an epigenetic factor to have utility as a biomarker or to facilitate early intervention, it must be detectable in peripheral samples. Secondly, despite the size and high longitudinal retention of the ARIES subsample (*n*~1000), numbers in the current analyses were reduced by the availability of both biological and phenotypic data. Previous research in ALSPAC has demonstrated non-random attrition, whereby participants with higher polygenic risk scores for schizophrenia were less likely to complete questionnaires and attend data collection sessions^53^, which may have reduced the power to detect factors associated with psychosis-related phenotypes in this study. Thirdly, it is difficult to identify the biological plausibility of these results, especially given the small differences detected which may represent technical artefacts rather than variation attributable to differences in PEs. However, given the focus here on pre-clinical symptoms of a complex psychiatric phenotype, it is to be expected that relatively small differences in DNAm would be observed, likely reflecting many genes of small effect. In addition, the scale of genome-wide epigenetic profiling techniques means that stringent multiple testing correction is required when interpreting the results. In this study, no CpG site in the EWAS analyses reached Bonferroni-corrected levels of significance. However, we were able to replicate one association (cg16459265) in an independent sample and observed effects in related gene sets such as *PTPRN2 and HLA-DPB2* in other studies. DNAm profiles across the array are not independent, and therefore adherence to strict multiple-testing thresholds, particularly in complex phenotypes, may lead to disregarding potentially interesting findings. Finally, only one of the CpG sites associated with PEs in the ALSPAC sample was replicated in the independent E-Risk sample. However, this may be due to methodological differences in the samples, such as the differences in timings between DNA sample collection and the data collection for PEs (DNA sample collection preceded the measurement of PEs in ALSPAC; in E-Risk they were collected concurrently).

## Conclusions

In conclusion, we found that DNAm across childhood and adolescence may be associated with the emergence and continuity of PEs between the ages of 12 and 18. Previous research has identified epigenetic patterns related to clinical diagnoses of psychotic disorders^15–22^, although to date very few have focussed on pre-clinical symptoms^23,24^ and thus the current findings substantially extend this literature. Research uncovering early biomarkers associated with PEs is important as it may, in the future, have the potential to facilitate early identification of individuals at increased risk of a range of mental health problems, and facilitate targeting of preventive interventions. Considered together with previous research, our findings provide tentative evidence for potential methylomic changes across timepoints spanning the development of early psychotic phenomena.

## Supplementary information

Supplementary Methods & Results

Supplementary Tables
