# Supplementary material for: Longitudinal investigation of DNA methylation changes preceding adolescent psychotic experiences
Source: Transl Psychiatry. 2019 Feb 4;9:69. doi: 10.1038/s41398-019-0407-8 (PMC6361958; doi:10.1038/s41398-019-0407-8)
Supplement: Supplementary file 1 — Supplementary Methods & Results [file 41398_2019_407_MOESM1_ESM.docx]

**Supplementary Information**

**Methods**

*Psychotic experiences*

Psychotic experiences at ages 12 and 18 were assessed via a semi-structured interview (PLIKSi) that has been described in detail previously (1, 2). At 12 years participants were asked about psychotic experiences over the previous 6 months, and at 18 years participants were asked about experiences since the age of 12. Participants were asked 11 core questions eliciting key psychotic experiences, covering hallucinations (visual and auditory), delusions (spied on, persecution, thoughts read, reference, control, grandiosity), and experiences of thought interference (broadcasting, insertion and withdrawal) as well unspecified delusions. ‘Maybe’ or ‘yes’ responses were followed by cross-questioning to establish whether experiences were psychotic and coding followed glossary definitions and rating rules for the Schedules for Clinical Assessment in Neuropsychiatry (SCAN; World Health Organisation) (3). At regular intervals, a psychiatrist rated samples of recorded interviews to ensure that the interviewers were rating experiences correctly. Each experience was rated by the interviewer as either: not present, suspected, or definitely present. The average Kappa values for inter-rater reliability were 0.72 and 0.83 for the 12-year and 18-year interviews, respectively. Test-retest agreement at 18 years was 0.74. In order to maximise variability in the outcome variable, psychotic experiences rated as either suspected or definite at ages 12 and 18 years were included regardless of whether or not they were attributable to sleep, fever or substance use.

**References**

1. Horwood J, et al. IQ and non-clinical psychotic symptoms in 12-year-olds: results from the ALSPAC birth cohort. *Br J Psychiatry* 2008; **193(3)**: 185-191.

2. Zammit S, et al. Psychotic experiences and psychotic disorders at age 18 in relation to psychotic experiences at age 12 in a longitudinal population-based cohort study. *Am J Psychiatry* 2013; **170(7)**: 742-750.

3. World Health Organisation. *Schedules for Clinical Assessment in Neuropsychiatry Version 2.0*. Washington, DC; 1994.

**Supplementary Table S15. Longitudinal analyses – trajectories of DNA methylation and continuity of psychotic experiences groups**

| **Probe ID** | **Annotated gene** | **PE trajectory** | **Estimate** | **SE** | **full model** | |
| --- | --- | --- | --- | --- | --- | --- |
|  |  |  |  |  | **t value** | **p value** |
| cg20862283 | - | PE - remission | 0.00844 | 0.0035 | 2.41 | 0.01604 |
|  |  | PE - emergence | -0.00478 | 0.00423 | -1.13 | 0.25917 |
|  |  | PE - persistence | 0.00524 | 0.00725 | 0.72 | 0.47052 |
| cg10490202 | RPTOR | PE - remission | -0.00159 | 0.000935 | -1.7 | 0.08896 |
|  |  | PE - emergence | -0.0011 | 0.00113 | -0.97 | 0.33168 |
|  |  | PE - persistence | -0.0067 | 0.00194 | -3.46 | 5.77E-04 |
| cg21040096 | RPH3AL | PE - remission | 0.0158 | 0.00901 | 1.75 | 0.08057 |
|  |  | PE - emergence | 0.00212 | 0.0109 | 0.19 | 0.84655 |
|  |  | PE - persistence | 0.00736 | 0.0188 | 0.39 | 0.69541 |
| cg18752363 | C4orf10; | PE - remission | -0.00675 | 0.00141 | -4.77 | 2.23E-06 |
|  | NOP14 | PE - emergence | 0.00229 | 0.00171 | 1.34 | 0.18106 |
|  |  | PE - persistence | -0.00244 | 0.00293 | -0.83 | 0.40622 |
| cg11271415 | FBXO32 | PE - remission | 0.0069 | 0.00209 | 3.31 | 0.00099 |
|  |  | PE - emergence | 0.00239 | 0.00252 | 0.95 | 0.3428 |
|  |  | PE - persistence | 0.00767 | 0.00432 | 1.77 | 0.07636 |
| cg20083186 | - | PE - remission | 0.00551 | 0.00228 | 2.42 | 0.0156 |
|  |  | PE - emergence | -2.57E-05 | 0.00275 | -0.01 | 0.99254 |
|  |  | PE - persistence | 0.00498 | 0.00472 | 1.05 | 0.29169 |
| cg00712792 | SPIRE2 | PE - remission | -0.000456 | 0.00207 | -0.22 | 0.82574 |
|  |  | PE - emergence | 0.00272 | 0.00251 | 1.09 | 0.278 |
|  |  | PE - persistence | 0.00137 | 0.0043 | 0.32 | 0.74932 |
| cg11936556 | - | PE - remission | 0.00706 | 0.00275 | 2.57 | 0.01037 |
|  |  | PE - emergence | -0.00515 | 0.00332 | -1.55 | 0.12163 |
|  |  | PE - persistence | 0.00424 | 0.0057 | 0.74 | 0.45674 |
| cg14892222 | GOLGA8A | PE - remission | 0.00764 | 0.00394 | 1.94 | 0.05307 |
|  |  | PE - emergence | 0.00235 | 0.00477 | 0.49 | 0.62218 |
|  |  | PE - persistence | 0.013 | 0.00817 | 1.6 | 0.11113 |
| cg26672652 | PXDNL | PE - remission | 0.00405 | 0.00308 | 1.31 | 0.18908 |
|  |  | PE - emergence | -0.002 | 0.00373 | -0.54 | 0.59221 |
|  |  | PE - persistence | -0.00115 | 0.00639 | -0.18 | 0.85693 |
| cg00407329 | SIM1 | PE - remission | 0.00216 | 0.00456 | 0.47 | 0.63669 |
|  |  | PE - emergence | 0.0183 | 0.00553 | 3.31 | 0.00097 |
|  |  | PE - persistence | 0.0217 | 0.00951 | 2.28 | 0.02288 |
| cg17972930 | USP50 | PE - remission | 0.00185 | 0.00333 | 0.56 | 0.5778 |
|  |  | PE - emergence | 0.0103 | 0.00402 | 2.55 | 0.01072 |
|  |  | PE - persistence | 0.00374 | 0.00689 | 0.54 | 0.58793 |
| cg07571954 | C10orf131 | PE - remission | 0.000155 | 0.00201 | 0.08 | 0.9387 |
|  |  | PE - emergence | -0.00512 | 0.00244 | -2.1 | 0.03568 |
|  |  | PE - persistence | -0.00141 | 0.00418 | -0.34 | 0.73585 |
| cg05138918 | - | PE - remission | 0.00373 | 0.00379 | 0.99 | 0.32494 |
|  |  | PE - emergence | -0.0107 | 0.00459 | -2.33 | 0.02015 |
|  |  | PE - persistence | -0.00718 | 0.0079 | -0.91 | 0.36356 |
| cg17843418 | TBC1D16 | PE - remission | 0.00643 | 0.00303 | 2.12 | 0.03401 |
|  |  | PE - emergence | -0.00978 | 0.00366 | -2.67 | 0.0077 |
|  |  | PE - persistence | 0.000804 | 0.00627 | 0.13 | 0.89806 |
| cg21167905 | C1orf9 | PE - remission | 0.00628 | 0.00362 | 1.73 | 0.08294 |
|  |  | PE - emergence | 0.0125 | 0.00438 | 2.85 | 0.00448 |
|  |  | PE - persistence | 0.00751 | 0.0075 | 1 | 0.31707 |
| cg09825080 | - | PE - remission | -0.00302 | 0.00264 | -1.14 | 0.25329 |
|  |  | PE - emergence | -0.00134 | 0.00319 | -0.42 | 0.67384 |
|  |  | PE - persistence | -0.0125 | 0.00547 | -2.28 | 0.02264 |
| cg02424103 | - | PE - remission | -0.00228 | 0.00355 | -0.64 | 0.52069 |
|  |  | PE - emergence | 0.0138 | 0.00429 | 3.21 | 0.00138 |
|  |  | PE - persistence | 0.0102 | 0.00736 | 1.39 | 0.16475 |
| cg16426764 | LHPP | PE - remission | -0.0151 | 0.00767 | -1.97 | 0.04892 |
|  |  | PE - emergence | -0.0213 | 0.00929 | -2.29 | 0.02232 |
|  |  | PE - persistence | -0.0413 | 0.016 | -2.59 | 0.00981 |
| cg00680277 | TTC15 | PE - remission | 0.00514 | 0.00451 | 1.14 | 0.25505 |
|  |  | PE - emergence | 0.0097 | 0.00545 | 1.78 | 0.07577 |
|  |  | PE - persistence | 0.0154 | 0.00935 | 1.64 | 0.10107 |
| cg22499215 | - | PE - remission | -0.00059 | 0.00128 | -0.46 | 0.64383 |
|  |  | PE - emergence | 0.000212 | 0.00154 | 0.14 | 0.89082 |
|  |  | PE - persistence | 0.000554 | 0.00264 | 0.21 | 0.83392 |
| cg14263553 | - | PE - remission | -0.00173 | 0.00124 | -1.39 | 0.16388 |
|  |  | PE - emergence | 0.00277 | 0.0015 | 1.84 | 0.06528 |
|  |  | PE - persistence | -0.00084 | 0.00257 | -0.33 | 0.74416 |
| cg07366553 | SELO | PE - remission | -0.007217 | 0.00327 | -2.21 | 0.0275 |
|  |  | PE - emergence | -0.004034 | 0.00396 | -1.02 | 0.30787 |
|  |  | PE - persistence | 0.0011263 | 0.00678 | 0.17 | 0.86809 |
| cg09553452 | CDH5 | PE - remission | -0.00493 | 0.00219 | -2.25 | 0.02459 |
|  |  | PE - emergence | 0.00027 | 0.00265 | 0.1 | 0.91882 |
|  |  | PE - persistence | -0.00397 | 0.00454 | -0.87 | 0.38176 |
| cg02284814 | PPP1R12B | PE - remission | -0.004756 | 0.00389 | -1.22 | 0.22174 |
|  |  | PE - emergence | -0.008239 | 0.0047 | -1.75 | 0.08021 |
|  |  | PE - persistence | -0.002503 | 0.00807 | -0.31 | 0.75636 |
| cg17160666 | - | PE - remission | -0.00384 | 0.00242 | -1.58 | 0.11316 |
|  |  | PE - emergence | -0.00836 | 0.00293 | -2.86 | 0.00434 |
|  |  | PE - persistence | -0.000511 | 0.00502 | -0.1 | 0.91892 |
| cg17139666 | - | PE - remission | 0.0092 | 0.00488 | 1.88 | 0.05991 |
|  |  | PE - emergence | -0.00465 | 0.00592 | -0.79 | 0.43224 |
|  |  | PE - persistence | 0.0211 | 0.0102 | 2.08 | 0.0382 |
| cg08032135 | NRG1 | PE - remission | 0.00102 | 0.00109 | 0.93 | 0.35111 |
|  |  | PE - emergence | -0.00221 | 0.00132 | -1.68 | 0.09403 |
|  |  | PE - persistence | 0.00219 | 0.00226 | 0.97 | 0.33171 |
| cg04584761 | SUPT7L | PE - remission | -0.011 | 0.00599 | -1.84 | 0.06532 |
|  |  | PE - emergence | -0.0102 | 0.00724 | -1.41 | 0.15863 |
|  |  | PE - persistence | 0.000993 | 0.0124 | 0.08 | 0.93624 |
| cg10756647 | PSPH | PE - remission | 0.00127 | 0.00135 | 0.94 | 0.34816 |
|  |  | PE - emergence | -0.00181 | 0.00163 | -1.11 | 0.26953 |
|  |  | PE - persistence | 0.00471 | 0.00281 | 1.68 | 0.09406 |
| cg00995854 | CD5L | PE - remission | 0.000893 | 0.00567 | 0.16 | 0.87481 |
|  |  | PE - emergence | -0.0184 | 0.00685 | -2.69 | 0.00728 |
|  |  | PE - persistence | 0.00181 | 0.0118 | 0.15 | 0.87765 |
| cg13845105 | RPL6 | PE - remission | 0.00466 | 0.00393 | 1.18 | 0.23661 |
|  |  | PE - emergence | -0.00614 | 0.00475 | -1.29 | 0.19712 |
|  |  | PE - persistence | -0.0116 | 0.00816 | -1.43 | 0.15456 |
| cg27190398 | STUB1 | PE - remission | 0.007052 | 0.00639 | 1.1 | 0.27001 |
|  | JMJD8 | PE - emergence | -0.00372 | 0.00773 | -0.48 | 0.63029 |
|  |  | PE - persistence | -0.006387 | 0.0133 | -0.48 | 0.63006 |
| cg07143863 | - | PE - remission | 0.0032 | 0.00185 | 1.73 | 0.08374 |
|  |  | PE - emergence | -0.00343 | 0.00223 | -1.53 | 0.12536 |
|  |  | PE - persistence | -0.00116 | 0.00383 | -0.3 | 0.76149 |
| cg10468951 | C3orf45 | PE - remission | -0.001022 | 0.00319 | -0.32 | 0.74916 |
|  |  | PE - emergence | -0.007036 | 0.00386 | -1.82 | 0.06865 |
|  |  | PE - persistence | -0.001975 | 0.00662 | -0.3 | 0.76554 |
| cg05927274 | - | PE - remission | -0.000428 | 0.00298 | -0.14 | 0.8857 |
|  |  | PE - emergence | -0.00588 | 0.0036 | -1.63 | 0.10305 |
|  |  | PE - persistence | -0.00786 | 0.00619 | -1.27 | 0.20433 |
| cg09936919 | - | PE - remission | -0.00203 | 0.00235 | -0.87 | 0.38658 |
|  |  | PE - emergence | -0.00437 | 0.00284 | -1.54 | 0.12424 |
|  |  | PE - persistence | -0.00231 | 0.00487 | -0.47 | 0.63563 |
| cg15129144 | EPAS1 | PE - remission | 0.00093 | 0.00215 | 0.43 | 0.66612 |
|  |  | PE - emergence | -0.00571 | 0.00261 | -2.19 | 0.02868 |
|  |  | PE - persistence | 0.000897 | 0.00447 | 0.2 | 0.84112 |
| cg03332469 | - | PE - remission | -0.00187 | 0.00148 | -1.26 | 0.20663 |
|  |  | PE - emergence | 0.00532 | 0.00179 | 2.98 | 0.00301 |
|  |  | PE - persistence | 0.0094 | 0.00307 | 3.07 | 0.00226 |
| cg02743632 | HLX | PE - remission | 0.000631 | 0.00345 | 0.18 | 0.85504 |
|  |  | PE - emergence | -0.00202 | 0.00418 | -0.48 | 0.62906 |
|  |  | PE - persistence | -0.00216 | 0.00716 | -0.3 | 0.76273 |
| cg14284469 | STMN2 | PE - remission | 0.00198 | 0.00133 | 1.5 | 0.13528 |
|  |  | PE - emergence | -0.000258 | 0.0016 | -0.16 | 0.87217 |
|  |  | PE - persistence | 0.00354 | 0.00275 | 1.29 | 0.19851 |
| cg00956759 | - | PE - remission | -0.00642 | 0.00253 | -2.54 | 0.0113 |
|  |  | PE - emergence | 0.00131 | 0.00306 | 0.43 | 0.66816 |
|  |  | PE - persistence | -0.00956 | 0.00524 | -1.82 | 0.06864 |
| cg12009697 | - | PE - remission | -0.00819 | 0.00371 | -2.21 | 0.02762 |
|  |  | PE - emergence | -0.00498 | 0.00449 | -1.11 | 0.2672 |
|  |  | PE - persistence | 0.00221 | 0.0077 | 0.29 | 0.77397 |
| cg21602768 | ABHD12 | PE - remission | -0.00116 | 0.00127 | -0.91 | 0.36113 |
|  |  | PE - emergence | 0.0024 | 0.00153 | 1.57 | 0.11709 |
|  |  | PE - persistence | -0.002 | 0.00262 | -0.76 | 0.44584 |
| cg17027353 | - | PE - remission | 0.0103 | 0.00409 | 2.51 | 0.01237 |
|  |  | PE - emergence | 0.0103 | 0.00494 | 2.08 | 0.03792 |
|  |  | PE - persistence | -0.00465 | 0.00848 | -0.55 | 0.58341 |
| cg14386808 | - | PE - remission | -0.003502 | 0.00355 | -0.99 | 0.3239 |
|  |  | PE - emergence | -0.004598 | 0.00429 | -1.07 | 0.28428 |
|  |  | PE - persistence | -0.01664 | 0.00736 | -2.26 | 0.02417 |
| cg22035229 | MSH4 | PE - remission | -0.0114 | 0.00798 | -1.43 | 0.15388 |
|  |  | PE - emergence | 0.00369 | 0.00966 | 0.38 | 0.70292 |
|  |  | PE - persistence | -0.0203 | 0.0166 | -1.22 | 0.2222 |
| cg16817992 | KCNH2 | PE - remission | 0.0113364 | 0.00468 | 2.42 | 0.01559 |
|  |  | PE - emergence | 0.0003978 | 0.00566 | 0.07 | 0.944 |
|  |  | PE - persistence | 0.010381 | 0.00973 | 1.07 | 0.28617 |
| cg25344401 | FOXK1 | PE - remission | 0.0059 | 0.0058 | 1.02 | 0.30959 |
|  |  | PE - emergence | 0.00661 | 0.00701 | 0.94 | 0.34585 |
|  |  | PE - persistence | 0.00362 | 0.012 | 0.3 | 0.7635 |
| cg05942128 | HOXD11 | PE - remission | 0.00179 | 0.00164 | 1.09 | 0.27554 |
|  |  | PE - emergence | -0.00131 | 0.00198 | -0.66 | 0.50885 |
|  |  | PE - persistence | 0.00321 | 0.00339 | 0.95 | 0.34477 |
| cg25975712 | FAM19A5 | PE - remission | 0.000934 | 0.00066 | 1.41 | 0.15763 |
|  |  | PE - emergence | 0.00251 | 0.000799 | 3.14 | 0.00175 |
|  |  | PE - persistence | 0.00373 | 0.00137 | 2.72 | 0.00663 |
| cg24177611 | KNDC1 | PE - remission | 0.0037 | 0.00571 | 0.65 | 0.51751 |
|  |  | PE - emergence | -0.00449 | 0.00691 | -0.65 | 0.51613 |
|  |  | PE - persistence | -0.031 | 0.0119 | -2.61 | 0.00915 |
| cg25324164 | FADS2 | PE - remission | -0.00306 | 0.00541 | -0.57 | 0.57187 |
|  |  | PE - emergence | -0.0128 | 0.00655 | -1.96 | 0.05038 |
|  |  | PE - persistence | -0.0197 | 0.0113 | -1.75 | 0.08093 |
| cg15986671 | - | PE - remission | 0.0073 | 0.00276 | 2.65 | 0.00827 |
|  |  | PE - emergence | -0.00757 | 0.00333 | -2.27 | 0.02343 |
|  |  | PE - persistence | -0.00123 | 0.00572 | -0.22 | 0.82917 |
| cg16073378 | - | PE - remission | 0.000956 | 0.00125 | 0.77 | 0.44422 |
|  |  | PE - emergence | 0.00251 | 0.00151 | 1.66 | 0.09756 |
|  |  | PE - persistence | -0.00113 | 0.00259 | -0.44 | 0.66298 |
| cg04942547 | ZFP64 | PE - remission | -0.00615 | 0.00249 | -2.47 | 0.01365 |
|  |  | PE - emergence | -0.00916 | 0.00301 | -3.05 | 0.00241 |
|  |  | PE - persistence | -0.00807 | 0.00516 | -1.56 | 0.11845 |
| cg11333576 | SHC2 | PE - remission | 0.00234 | 0.00253 | 0.92 | 0.35544 |
|  |  | PE - emergence | -0.0048 | 0.00306 | -1.57 | 0.11724 |
|  |  | PE - persistence | -0.00971 | 0.00525 | -1.85 | 0.06433 |
| cg16459265 | C7orf40 | PE - remission | 0.00783 | 0.00627 | 1.25 | 0.2118 |
|  | SNORA9 | PE - emergence | -0.0179 | 0.0076 | -2.36 | 0.01878 |
|  |  | PE - persistence | -0.0285 | 0.0131 | -2.18 | 0.02967 |
| cg09222367 | IQGAP1 | PE - remission | -0.000352 | 0.00299 | -0.12 | 0.90628 |
|  |  | PE - emergence | -0.00737 | 0.00362 | -2.04 | 0.04176 |
|  |  | PE - persistence | 0.00201 | 0.00621 | 0.32 | 0.74628 |
| cg23415756 | NTN1 | PE - remission | -0.000421 | 0.000348 | -1.21 | 0.22663 |
|  |  | PE - emergence | 0.000108 | 0.000421 | 0.26 | 0.79707 |
|  |  | PE - persistence | 0.0000758 | 0.000721 | 0.11 | 0.9163 |

*PE – psychotic experiences; Remission – PEs at 12 but not 18; Emergence – PEs at 18 but not 12; Persistence – PEs at both time-points; Light grey cells: p<0.05; Dark grey cells: p<0.00045.*
